# Molecular Identification of *Borreliella* Species in *Ixodes hexagonus* Ticks Infesting Hedgehogs (*Erinaceus europaeus* and *E. roumanicus*) in North-Western Poland

**DOI:** 10.3390/ijms26010058

**Published:** 2024-12-25

**Authors:** Kolomiiets Valentyna, Wodecka Beata

**Affiliations:** 1 Department of Genetics and Genomics, Institute of Biology, University of Szczecin, Wąska 13, 71-415 Szczecin, Poland; valentyna.kolomiiets@usz.edu.pl; 2 Doctoral School, University of Szczecin, Adama Mickiewicza 16, 70-384 Szczecin, Poland

**Keywords:** hedgehogs, zoonoses, infections, *Borreliella*

## Abstract

The western European hedgehog (*Erinaceus europaeus*) and the northern white-breasted hedgehog (*E. roumanicus*) are natural hosts of the tick *Ixodes hexagonus*, the vector of tick-borne pathogens such as the *Borreliella* bacteria responsible for Lyme disease. The aim of this study was to identify these pathogens in ticks collected from hedgehogs in northwestern Poland and to assess their genetic diversity by molecular analysis of the detected pathogens based on the *flaB* gene and the *mag-trnI* intergenic spacer. Among 101 hedgehogs examined, 737 ticks were found on 56 (55.45%) individuals, including 501 females of *I. hexagonus*. *Borreliella* spirochete infection was confirmed in 9 females of *I. hexagonus* (1.8%) obtained from 4 (3.96%) hedgehogs, detecting *Borreliella (Bl.) afzelii* (8/89%) and *Bl. spielmanii* (1/11%). Phylogenetic analysis based on the *flaB* gene and the *mag-trnI* intergenic spacer showed a lack of diversity in *Bl. afzelii* detected in *I. hexagonus* ticks collected from hedgehogs as well as little diversity against reference strains detected in small mammals and ticks collected from them. The results confirm that hedgehogs play an important role in the circulation of the detected spirochete species, at least as hosts of *I. hexagonus* ticks infected with them, indicating their potential to spread *Borreliella* spirochetes.

## 1. Introduction

Hedgehogs are widespread insectivorous mammals that play a key role in the ecosystems of Europe. They are quite common in synanthropic environments such as gardens, cemeteries, city parks, and urban backyards. They usually forage within 200–300 m from their nests, which is their home range, but sometimes they can travel distances of several kilometers [1]. Due to their close contact with human habitats and their ability to transmit a variety of enteric (fecal-borne) and vector-borne (transmitted by blood-sucking arthropods) pathogens, hedgehogs can be an important reservoir of zoonotic diseases [2]. In recent years, there has been increased interest in the study of pathogens carried by these animals, in particular, tick-borne bacteria of the genus *Borreliella* [3,4,5,6,7,8]. Two species of these animals are found in Poland: the western European hedgehog (*Erinaceus europaeus*) and the northern white-breasted hedgehog (*E. roumanicus*).

Tick-transmitted *Borreliella* bacteria are spirochetes known as the etiological agents of Lyme borreliosis. In Poland, as in other European countries, this disease is a serious health problem [9]. Therefore, it is important to understand the role of hedgehogs as potential reservoirs of these pathogens and to determine the extent of their prevalence in hedgehog populations. The European hedgehog (*E. europaeus*) is known to be involved in the enzootic cycle of *Borreliella* and *Anaplasma phagocytophilum* bacteria. These microorganisms are transmitted by ticks of the genus *Ixodes,* such as *I. ricinus* and *I. hexagonus,* which are both found in hedgehogs in Central Europe [10,11]. Moreover, *Haemaphysalis erinacei*, *Rhipicephalus turanicus*, and *Rh. sanguineus* sensu lato have also been found on European hedgehogs in the southern regions of their range [12]. The prevalence of *Borreliella species* and *A.phagocytophilum*, the most common pathogens found in ticks collected from hedgehogs, varies geographically [13,14]. The role of hedgehogs in the epidemiology of tick-borne pathogen infections is not fully recognized; nevertheless, given the increasing number of hedgehogs (and ticks carried by them) in the human environment caused by anthropogenic activity [15], further research focused on these mammals is certainly needed.

The aim of the research is the molecular identification of spirochetes of the *Borreliaceae* family in ticks infesting hedgehogs (*Erinaceus romanicus* and *E. europaeus*) from rescue services located in northwestern Poland.

This study can help raise awareness of the importance of hedgehogs as reservoirs of pathogens and help develop effective strategies to prevent and control the spread of diseases carried by these animals.

## 2. Results

### 2.1. Morphological Identification of Ticks

In the period 2022–2024, 101 rescued hedgehogs were examined for the presence of ticks. 56 animals (55.45%) carried ticks, which were collected for a total of 737 specimens belonging to *Ixodes* sp. (Table 1).

Of the 505 adult ticks collected (Table 1), 4 represented the species *I. ricinus* and 501 *I. hexagonus*. The analysis of feeding revealed that all 4 *I. ricinus* specimens collected were fully engorged. For infected *I. hexagonus* ticks, one unfed female, 5 fully engorged females and 3 partially engorged females were identified. All collected males of *I. hexagonus* were unfed. Analysis of feeding status of all collected females of *I. hexagonus* showed that 298 of them were fully engorged, 165 were partially engorged and 30 were unfed. In addition, all collected nymphs and larvae were fully engorged.

**Figure 1:**
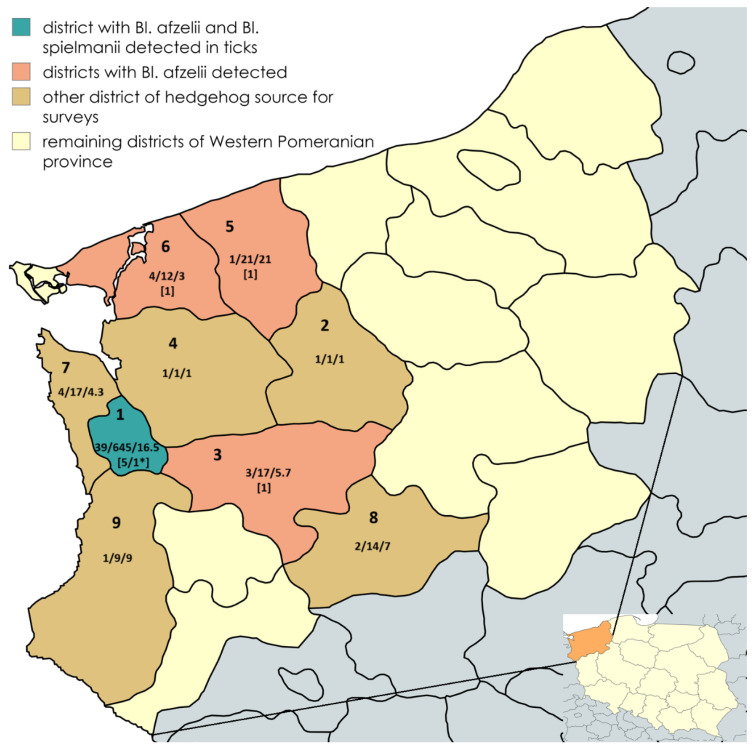
Location of retrieval sites for hedgehogs *Erinaceus* sp in Western Pomeranian Province, Poland: 9 districts: (1) Szczecin District, 53°26′17″ N 14°32′32″ E—city of Szczecin, (2) Łobez District: 53°32′20″ N 15°33′46″ E—Węgorzyno town, (3) Stargard District: 53°20′15″ N 15°02′16″ E—Stargard town, (4) Goleniów County: 53°29′46,79″ N 15°03′41,12″ E—Maszewo town, (5) Gryfice District: 53°54′24″ N 15°22′03″ E—Natolewice village, (6) Kamień Pomorski District: 53°50′29″ N 14°36′41″ E—Wolin town, 53°58′08″ N 14°46′45″ E—Kamień Pomorski town, (7) Police District: 53°39′26″ N 14°30′38″ E—Trzebież village, 53°25′48″ N 14°27′58″ E—Mierzyn village, 53°26′07″ N 14°24′32″ E—Dołuje village, (8) Choszczno District: 53°10′09″ N 15°25′10″ E—Choszczno town, 53°06′23″ N 15°24′25″ E—Zwierzyn village, (9) Gryfino District: 52°52′45″ N 14°12′08″ E—Cedynia town, 52°46′16″ N 14°33′40″ E—Troszyn village. Numbers listed are number of hedgehogs infested/number of ticks/average number of ticks per hedgehog. In square brackets: number of infected ticks and so the number of hedgehogs carrying a tick infected with *Bl. afzelii*/* *Bl. spielmanii*.

The Szczecin district recorded the highest abundance of infested hedgehogs (39 of 56/69.6%) and tick prevalence (645/87.5%, Figure 1). Districts such as Stargard, Goleniów, and Kamień Pomorski are characterized by lower abundance, and varying average numbers of ticks per host (Figure 1), which may indicate local differences in parasite density and activity in hedgehog shelter sites.

### 2.2. Detection of Borreliaceae DNA and Idetification of Species

DNA of spirochetes of the *Borreliaceae* family was detected on the basis of nested PCR reactions with primers specific for the *flaB* gene sequence and the *mag* and *trnI* genes surrounding a non-coding region of variable sequence length in 9 (1.8%) out of 501 adult *I. hexagonus* specimens, representing 1.2% of the total study population of 737 ticks collected from hedgehogs. All infected individuals were females of *I. hexagonus*, and each was collected from a separate hedgehog. No spirochete DNA was detected in female *I. ricinus* ticks, and no infections were found in other males of *I. hexagonus* and in other stages of *Ixodes* sp. A comparison of the degree of infection in ticks representing different life stages, as well as in females representing the two morphologically identified species, showed no statistically significant differences (*p* > 0.2).

Using the species-specific sequence length polymorphism occurring between the *mag* and *trnI* genes, it was possible to identify two species of the genus *Borreliella* based on the characteristic lengths of the PCR products: 793 bp for *Bl. afzelii* and 498 bp for *Bl. spielmanii*. *Borreliella afzelii* was detected in eight samples and *Bl. spielmanii* in one sample.

### 2.3. Analysis of the Genetic Variability of Borreliaceae Spirochetes

Analysis of the six sequences of the *flaB* gene fragments obtained in the study and comparison with reference strains confirmed species identity consistent with that obtained on the basis of the sequence length polymorphism occurring between the *mag* and *trnI* genes using the nested PCR technique. The mean genetic distance within the identified spirochete species was 0.001 for *Bl. spielmanii* and 0.008 for *Bl. afzelii*.

The interspecies genetic distance, calculated by comparing the sequences of *Bl. afzelii* and *Bl. spielmanii*, was 0.0531 and was within the range of genetic distance found between other *Borreliella* species present in Europe ranging from 0.0203 to 0.0763 (Table S1). These results show significantly higher diversity values than the intra-species variation values obtained for both *Borreliella* species detected in this study, confirming their correct species identification.

Analysis of nine sequence fragments specific to the region between the *mag* and *trnI* genes and their comparison with reference strains confirmed the species identity of the pathogens detected using the nested PCR technique. The average genetic distance within the identified vertebrate species was 0.0059 for *Bl. afzelii* and 0.0245 for *Bl. spielmanii*. These results indicate a low level of intra-species variability, which is characteristic of sequences belonging to the same species.

The interspecies genetic distance, calculated by comparing the sequences of *Bl. afzelii* and *Bl. spielmanii*, was 0.1086 and was within the range of values found for interspecies distances between the other species present in Europe, which ranged from 0.0528 (between *Bl. burgdorferi* and *Bl. finlandensis*) to 0.2548 (between *Bl. bissettiae* and *Bl. valaisiana*) (Table S2). These values also show significantly higher interspecies variation compared to intraspecies variation, confirming the correctness of the species identification of the spirochetes detected in the study.

**Figure 2:**
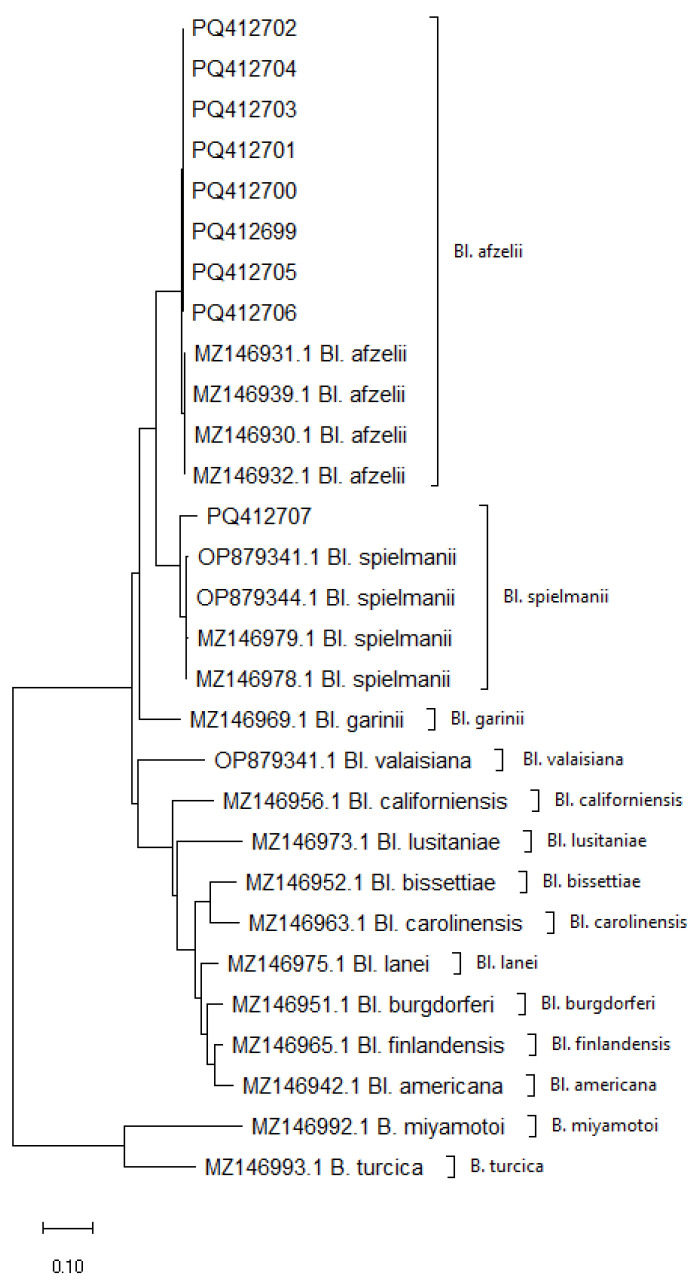
Phylogenetic analysis of *Borreliaceae* species derived from the *mag-trnI* intergenic spacer amplified using primers mag-435F and trnI-65R. The sequences with accession numbers PQ4126990-PQ412707 were obtained in this study.

**Figure 3:**
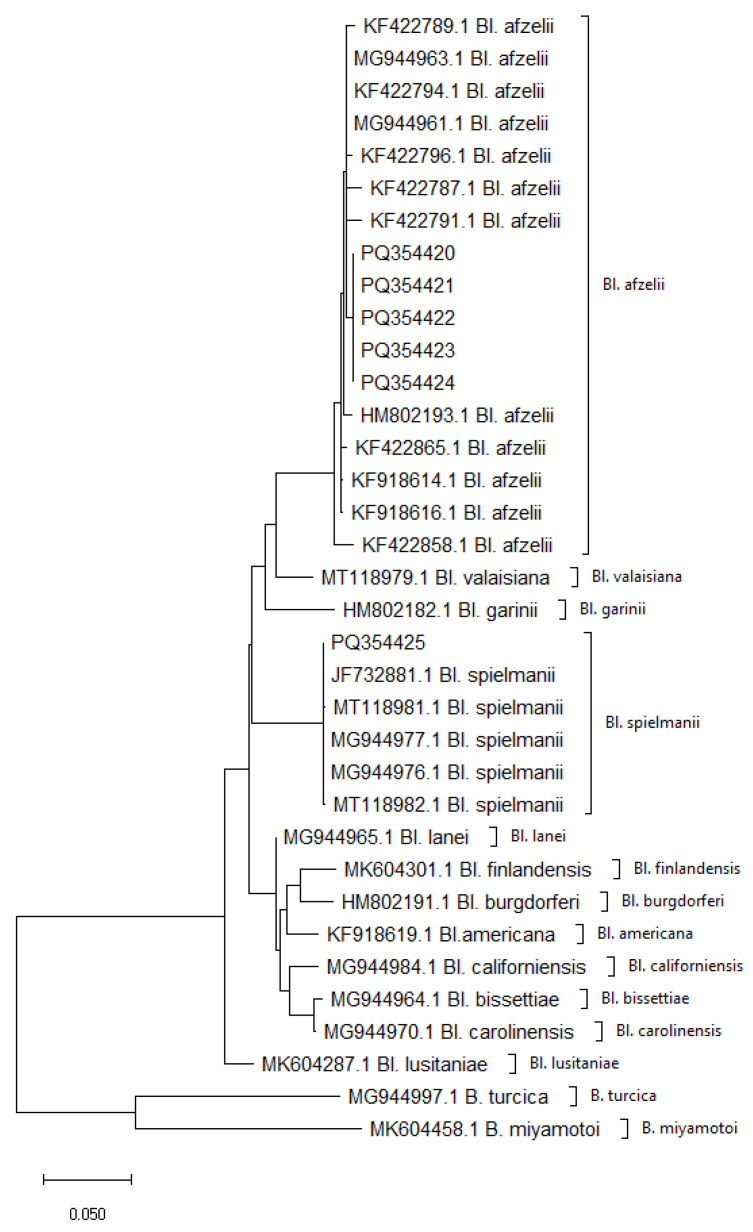
Phylogenetic analysis of *Borreliaceae* species derived from the *flaB* gene fragment amplified with primers FL220F and FL823R. The sequences with accession numbers PQ354420-PQ354425 were obtained in this study.

Phylogenetic analysis based on the *mag-trnI* intergenic sequence and the *flaB* gene sequence confirmed the identity of each of the species studied, as they clustered separately from each other, forming independent branches on both dendrograms obtained from the genetic markers analyzed (Figure 2 and Figure 3).

The results obtained from both analyses show a clear distinctiveness of the species within the genus *Borreliella*, which clearly indicates their phylogenetic relationships.

Both analyses showed concordance in the grouping of individual species, suggesting that both the genetic marker *flaB* and the intergenic spacer *mag-trnI* are suitable for phylogenetic analysis and classification of species in the genus *Borreliella*. The well-separated groupings on the phylogenetic trees demonstrate the stability of these markers and their high suitability for distinguishing closely related species.

## 3. Discussion

European hedgehogs (*E. europaeus*) and eastern hedgehogs (*E. roumanicus*) are widespread insectivorous mammal species that play an important role in ecosystems as consumers of invertebrates. Their biology, food, and habitat make them found in areas close to human settlements, including parks, gardens, and suburban areas. This proximity promotes hedgehog interactions with humans and pets, which can lead to a direct transmission of ectoparasites [16]. The ticks *I. hexagonus* and *I. ricinus* play a role as vectors for pathogens transmitted to humans and pets, including *Borreliella* bacteria (comprising *Bl. afzelii* and *Bl. spielmanii*), which cause Lyme borreliosis. Studies have shown that hedgehogs are important hosts for these ticks, which can lead to secondary cycles of pathogen transmission in urban environments, even if direct contact with humans is rare [16,17]. It is worth noting that the different life stages of ticks (larvae, nymphs, adults) may play different roles in pathogen transmission, and their occurrence often depends on local environmental conditions and host availability [18,19].

Tick occurrence not only affects the health of hedgehogs, but also poses a potential threat to public health, especially in urban and peri-urban areas, where the prevalence of pathogens is often underestimated [20].

A total of 737 ticks were collected in the study, of which 501 adults were identified as *I. hexagonus*, making this species the dominant one in the hedgehog samples. The analysis carried out in different districts of the Western Pomeranian Province showed that the largest number of ticks (645, 87.5%) were collected in Szczecin District, where the largest number of infested hedgehogs was also recorded (39, 69.6%). The average number of ticks per host ranged from 1.0 in the Goleniów district to 21.0 in the Gryfice district, indicating significant differences in infestation between regions. It is worth noting that *I. hexagonus* is a typical ectoparasite associated with hedgehogs, which explains its numerical predominance. Other studies also confirmed the dominance of this species on hedgehogs in Europe, where, in different regions, its proportion ranged from 50% [3,20]. In contrast, *I. ricinus*, being a generalist species, prefers more and habitats, which explains its lower abundance on hedgehogs. Of all ticks collected, 9 (1.22%) were infected with spirochetes of the genus *Borreliella*, of which 8 showed the presence of *Bl. afzelii* and 1 *Bl. spielmanii*, which are typically associated with small but also larger mammals [3,18]. *Bl. afzelii* and *Bl. spielmanii* infections are typically associated with small mammals such as rodents (e.g., voles) and small carnivores, and interspecies transmission of ticks and pathogens can also occur to mammalian predators such as foxes, raccoons, badgers, or martens [18,21]. *Bl. spielmanii* has mainly been identified in small mammals such as dormice, but also larger mammals can carry the bacteria, such as foxes [18].

The study showed that *Borreliella* spirochete infection occurred only in females of *I. hexagonus*, which may be the main vector for transmitting *Bl. afzelii* and *Bl. spielmanii* to these mammals, sustaining their further circulation in urban and suburban environments. The high density of these ticks on hedgehogs is due to the habitat preference for temporary nesting of both dominant species in Europe, which inhabit sites close to human habitation such as gardens, parks or suburban areas [16,21]. Other studies, conducted in Germany and Switzerland, have found a similar pattern of hedgehog infestation by ticks and transmission of *Borreliella* pathogens, suggesting a consensus of these findings on a wider geographical scale [3,20].

The phylogenetic analysis carried out in this study revealed low genetic variability in the detected *Borreliella* species, which is consistent with observations for other pathogens associated with small mammals. The low genetic variability is due to the limited range of mobility of hedgehogs, resulting in the local circulation of pathogens in a relatively homogeneous environment [19]. This is typical for pathogens associated with small mammals, where the low number of hosts and their limited mobility reduce gene exchange between different bacterial populations, resulting in lower genetic variability [22,23]. In particular, *Bl. afzelii*, as a species mainly associated with small mammals, shows low genetic variability compared to other pathogens associated with more mobile hosts such as birds [19,24]. Similarly to *Bl. afzelii*, the low genetic variability of *Bl. spielmanii* is due to the limited mobility of specific hosts, leading to local circulation of pathogens in relatively homogeneous host populations. Such results are typical for pathogens with narrow specificity to mammals [25].

From an epidemiological point of view, our results suggest that hedgehogs may play a role as reservoirs of pathogens of the genus *Borreliella*, in particular the species *Bl. afzelii*. This species appears to be the main pathogen associated with these mammals in north-western Poland. The results are consistent with other studies that also showed the dominance of *Bl. afzelii* in tick populations of *I. hexagonus* collected from small mammals in Europe [7].

Despite the sympatric presence of the two hedgehog species in the study area, genetic variation analysis showed that their presence does not affect the genetic variation in *Bl. afzelii*, suggesting that this spirochete species circulates mainly within homogeneous environments. Such results may confirm the genetic stability of *Borreliella* species associated with small mammals in environments with low host population mobility [23].

## 4. Materials and Methods

### 4.1. Ticks and Hedgehogs Sampling

The ticks obtained for the study came from hedgehogs from the Szczecin Hedgehog Rescue Service ‘Hedgehogs’ operating in Szczecin, which cooperates with the Polish Hedgehog Protection Association ‘Our Hedgehogs’ and the Szczecin Branch of the Society for Animal Care. This rescue service, certified as Hedgehog Keeper No. 27 by the General Directorate of Environmental Protection (Warsaw, Poland), provides assistance to hedgehogs and collects biological material. Tick specimens were obtained within the framework of cooperation with the rescue service; it was possible to obtain specimens of ticks collected by employees of this institution.

The ticks for the study came from hedgehogs delivered to the hedgehog rescue service from nine districts located in the western part of the West Pomeranian Voivodeship: Szczecin, Stargard, Goleniów, Police, Gryfino, Choszczno, Kamień Pomorski, Łobez, and Gryfice. These areas are characterized by an abundance of forests preceded by urbanized areas, which is suitable for hedgehogs.

### 4.2. DNA Extraction

DNA extraction from ticks was performed using the GeneMATRIX Tissue and Bacterial DNA Purification Kit (EURx, Gdansk, Poland), according to the manufacturer’s protocol. Prior to the actual isolation procedure, all tick specimens (larvae, nymphs, and adults) were suspended in 100 µL of PBS buffer and crushed by shaking at 50 Hz for 5 min in Eppendorf tubes with steel beads using a TissueLyser LT homogenizer (Qiagen, Hilden, Germany).

### 4.3. Detection of DNA of Borreliaceae Spirochetes and Identification of Species

The nested PCR method, which allows for the amplification of specific DNA fragments with high efficiency and specificity, was used to detect *Borreliaceae* spirochete DNA. Two sets of primers were used to amplify fragments of the *flaB* gene and the region between the *mag* and *trnI* genes (Table 2). Species identification was performed using a nested PCR procedure based on the sequence length polymorphism of the PCR product obtained with primers mag-435F and trnI-65R as previously described [19].

In each PCR run, DNA from the *Bl. burgdorferi* IRS reference strain (German Collection of Microorganisms and Cell Cultures-DSMZ, Leibniz, Germany) was used as a positive control and TE buffer as a negative control. PCR products were separated on a 1.5% agarose gel [19].

### 4.4. DNA Sequencing of Borreliaceae Spirochetes and Genetic Variation Analysis

For the positive amplicons obtained, fragments of the *flaB* gene were sequenced and amplified using the FL220F/FL823R primer pair, as well as *mag-trnI* intergenic spacers obtained with mag-435f/trnI-65r primers specific for *Borreliaceae* spirochetes (Table 2). DNA sequencing was performed at Macrogen Europe (Amsterdam, The Netherlands).

The obtained sequences were aligned using MEGA11 software and applying the MUSCLE algorithm [26] for further genetic variation analyses. Further analyses included the assessment of genetic variation by calculating intra- and interspecific genetic diversity indices to identify phylogenetic relationships between different isolates. The obtained sequences of the *flaB* gene fragments and the *mag-trnI* intergenic spacer were compared with those of reference strains available in the GenBank database (Tables S3 and S4).

The relatedness between the analyzed individuals was assessed on the basis of genetic distances, which were estimated as a measure of the number of allelic substitutions of the analyzed fragments within the selected loci. This process consisted of calculating the number of nucleotide differences between sequences, divided by the total number of nucleotides compared. The genetic distances thus obtained were used to assess genetic diversity both within single species (intraspecies diversity) and between different species (interspecies diversity).

The MEGA11 software enabled the best DNA analysis model to be selected based on empirical data, ensuring high accuracy of the results. The calculation of genetic distances within the *flaB* gene was based on the Tamura 3-parameter model, which is suitable for the analysis of DNA sequences with moderate levels of variation [26]. This model was used in the maximum likelihood (ML) method to provide an accurate assessment of genetic relatedness. Bootstrap values, derived from 1000 replicates, were used to estimate the stability of the resulting phylogenetic trees.

For the analysis of the *mag-trnI* intergenic spacer, the Hasegawa-Kishino-Yano model was used, which better captures the specificity of the evolution of these DNA regions.

Sequences of the *flaB* gene and *mag-trnI* intergenic spacer of *Borreliaceae* spirochetes have been deposited in the GenBank database. The *flaB* gene sequences were deposited under accession numbers: PQ354420-PQ354424 (*Bl. afzelii*), PQ354425 (*Bl. spielmanii*). The sequences of the *mag-trnI* intergenic spacer deposited in the GenBank database are as follows: PQ412699-PQ412706 (*Bl. afzelii*), PQ412707 (*Bl. spielmanii*).

### 4.5. Statistical Analyses

Statistical analyses were performed to assess the variation in the prevalence of *Borreliaceae* spirochetes in tick populations and to test the significance of differences in the intensity of tick infestation. The prevalence of *Borreliaceae* spirochetes in tick samples was assessed using a chi-square test with Yates’ correction and using Fisher’s exact test to correct for potential errors due to small sample sizes or unequal distribution of data. The analysis accounted for differences in infection rates between different stages of tick development and between different species among female ticks. All statistical analyses were performed using Statistica software version 13.3 (StatSoft Inc., Tulsa, OK, USA).

### 4.6. Morphological Identification of Ticks

Morphological identification of ticks was carried out based on the morphological features of adult individuals, which is much easier compared to immature stages (nymphs and larvae), whose characteristic morphological features are more erased during the parasitic stage. Females and males were identified using taxonomic keys developed by Siuda [27] and Estrada-Peña et al. [28].

## 5. Conclusions

The results obtained indicate that hedgehogs play a key role as hosts for *I. hexagonus* ticks transmitting zoonotic pathogens, which has significant implications for public health, particularly in urban and suburban areas. At the same time, despite the significant number of ticks found on hedgehogs, which exceeds three times the number of ticks found on foxes in western Poland, the proportion of *Borreliella*-infected individuals was a small proportion of the total pool of ticks examined compared to the several times higher infection rate of ticks collected from foxes [18]. However, further monitoring of the role of hedgehogs in the *Borreliella* circulation cycle is needed, especially in the context of the observed climate change and its impact on the population dynamics of these pathogens. In the context of the role of hedgehogs in the transmission of pathogens, attention should be given to the need to diversify conservation efforts and monitor populations of these animals to reduce the risk of zoonotic disease transmission to humans [20].

## Figures and Tables

**Table 1:** Occurrence of *Ixodes* species collected from hedgehogs rescued in northwestern Poland.

Tick Species	Total N//n/$\bar{x}$	Females N//n/$\bar{x}$	Males N//n/$\bar{x}$	Nymphs N//n/$\bar{x}$	Larvae N//n/$\bar{x}$
*I. ricinus*	3/4/0.75	3/4/0.75	0	0	0
*I. hexagonus*	47/501/10.65	47/493/10.5	6/8/1.3	0	0
*Ixodes* sp.	24/232/9.6	0	0	22/194/8.8	7/38/5.4
Total	56/737/14.5	54/497/9.2	6/8/1.3	22/194/8.8	7/38/5.4

N—the number of hedgehogs infested, n—number of ticks collected, $\bar{x}$ —average number of ticks per infested host.

**Table 2:** Primers used for DNA amplification of spirochetes of the Borreliaceae family.

Marker	Primer Sequence 5′-3′	Amplicon Size (bp)	Reference
*flaB*	step I FL84F: AGAAGCTTTCTAGTGGGTACAGA FL976R: GATTGGCCTGTGCAATCAT step II FL220F: CAGACAACAGAGGGAAAT FL823R: TCAAGTCTATTTTGGAAAGCACC	604	[19]
*mag*/*trnI*	step I mag-268F: TCTAATTAAAACAGCHTGDGGAYT trnI-20R: TGAACATCCGACCTCAGG step II mag-435F: CCATATAAGCTTCCGTTTCAAC trnI-65R: CTAACCACCTGAGCTATGATCC	309–1183	

## Data Availability

All data generated or analyzed during this study are included in this published article and its Supplementary Information file. The accession numbers of DNA sequences obtained for ticks and bacteria are mentioned in Material and Methods and are available in the GenBank (https://www.ncbi.nlm.nih.gov/nuccore, 15 October 2024).

## Supplementary Materials

The following supporting information can be downloaded at: https://www.mdpi.com/article/10.3390/ijms26010058/s1.
