# Supplementary material for: Molecular Identification of Borreliella Species in Ixodes hexagonus Ticks Infesting Hedgehogs (Erinaceus europaeus and E. roumanicus) in North-Western Poland
Source: Int J Mol Sci. 2024 Dec 25;26(1):58. doi: 10.3390/ijms26010058 (PMC11719967; doi:10.3390/ijms26010058)
Supplement: Supplementary file 1 [file ijms-26-00058-s001.zip › ijms-3343322-supplementary.pdf]

Table S1. MEGA 11 results of mean distance between *Borreliaceae* species obtained on the basis of *flaB* gene sequence fragment comparison

	BA	BS	BB	BG	BV	BBI	BCL	BCR	BLN	BAM	BL	BTC	BM
B. spielmanii	0,05310												
Bl. burgdorferi	0,06413	0,06348											
Bl. garinii	0,06363	0,05808	0,06659										
Bl. valaisiana	0,04961	0,05993	0,06099	0,05578									
Bl. bissettiae	0,06173	0,06002	0,04128	0,05939	0,05019								
Bl. californiensis	0,06196	0,06189	0,03949	0,06126	0,04837	0,03255							
Bl. carolinensis	0,05437	0,05263	0,03770	0,05940	0,04295	0,00667	0,02901						
Bl. lanei	0,05277	0,04899	0,02713	0,04838	0,03938	0,02377	0,02203	0,02030					
Bl. amerikana	0,06331	0,06556	0,03587	0,05932	0,05195	0,04323	0,03427	0,03963	0,02199				
Bl. lusitaniae	0,06503	0,05817	0,05922	0,05755	0,05403	0,05210	0,05394	0,05210	0,03765	0,05756			
B. turcica	0,17786	0,18286	0,18233	0,18407	0,16056	0,17344	0,17130	0,16700	0,16279	0,16910	0,16473		
B. miyamotoi	0,16929	0,17226	0,18501	0,19160	0,16070	0,18256	0,17807	0,17358	0,16700	0,16909	0,16062	0,14697	
Bl. finlandensis	0,07633	0,07459	0,03410	0,07017	0,06269	0,04655	0,04475	0,04655	0,02880	0,03755	0,06092	0,18185	0,18910

BA – *Bl. afzelii*, BS – *Bl. spielmanii*, BB – *Bl. burgdorferi*, BG – *Bl garinii*, BV – *Bl. valaisiana*, BBI – *Bl. bissettiae*, BCL – *Bl. californiensis*, BCR – *Bl. carolinensis*, BLN – *Bl. lanei, BAM – Bl. americana, BL – Bl. lusitaniae, BTC – B. turcica, BM – B. miyamotoi, BF – Bl. finlandensis*.

Table S2. MEGA 11 results of mean distance between *Borreliaceae* species obtained on the basis of intergenic spacer of 3-methyladenine glycosylase (*mag*) and tRNA-Ile (*trnI*) genes sequence fragment comparison

	BA	BS	BG	BM	BB	BV	BL	BBI	BF	BCL	BCR	BLN	BAM
Bl. spielmanii	0,1086												
Bl. garinii	0,1598	0,1322											
B. miyamotoi	0,4058	0,4036	0,4283										
Bl. burgdorferi	0,2043	0,2085	0,1968	0,4068									
Bl. valaisiana	0,2070	0,1532	0,2219	0,3867	0,2029								
Bl. lusitaniae	0,2532	0,2113	0,2337	0,3983	0,1607	0,2196							
Bl. bissettiae	0,2492	0,2325	0,2209	0,4323	0,1324	0,2548	0,2036						

Bl. finlandensis	0,1943	0,1983	0,2004	0,4318	0,0528	0,1807	0,1399	0,1356					
Bl. californiensis	0,1712	0,1653	0,1322	0,4271	0,1255	0,1511	0,1409	0,1248	0,1171				
Bl. carolinensis	0,2225	0,2264	0,1799	0,4557	0,1083	0,2117	0,1660	0,0965	0,1005	0,1324			
Bl. lanei	0,1991	0,1705	0,1781	0,4254	0,0674	0,1776	0,1618	0,1305	0,0751	0,1043	0,1121		
Bl. americana	0,2221	0,2090	0,1887	0,4336	0,0864	0,1902	0,1515	0,1545	0,0574	0,1383	0,1299	0,0813	
B. turcica	0,3507	0,3812	0,3296	0,2603	0,3301	0,3655	0,3776	0,3288	0,3529	0,3600	0,3518	0,3529	0,3378

BA – *Bl. afzelii*, BS – *Bl. spielmanii*, BG – *Bl garinii,* BM – *B. miyamotoi,* BB – *Bl. burgdorferi*, BV – *Bl. valaisiana*, BL – *Bl. lusitaniae*, BBI – *Bl. bissettiae*, BF – *Bl. finlandensis*, BCL – *Bl. californiensis*, BCR – *Bl. carolinensis*, BLN – *Bl. lanei*, BAM – *Bl. americana*, BTC – *B. turcica.*

Table S3. Reference strains of *Borreliaceae* spirochetes used for the comparison of the *mag-trnI* intergenic spacer.

Borreliaceae Species	Strain	Source	Country	Accession Number
Bl. afzelii	CM853	*I. ricinus*	Poland	MZ146930
	CM915	*I. ricinus*	Poland	MZ146931
	CM950	*I. ricinus*	Poland	MZ146932
	BR1958	*I. ricinus*	Poland	MZ146939
	7N1N-IC	*I. canisuga* fed on *Vulpes vulpes*	Poland	MT119025
	28K1N-IH	*I. hexagonus* fed on *Vulpes vulpes*	Poland	MT119026
Bl. spielmanii	GD1646	*I. ricinus*	Poland	MZ146979
	LB1379	*I. ricinus*	Poland	MZ146978
	SW234-12	*I. ricinus*	Poland	OP879344
	SW66-12	*I. ricinus*	Poland	OP879341
Bl. garinii	SW217	*I. ricinus*	Poland	MZ146969
Bl. burgdorferi	BD2504	*I. ricinus*	Poland	MZ146951
Bl. valaisiana	SW60-12	*I. ricinus*	Poland	OP879338
Bl. lusitaniae	CM850	*I. ricinus*	Poland	MZ146973
Bl. bissettiae	ZL2273	*I. ricinus*	Poland	MZ146952
Bl. finlandensis	SW180	*I. ricinus*	Poland	MZ146965
Bl. californiensis	CM1034	*I. ricinus*	Poland	MZ146956
Bl. carolinensis	GD1645	*I. ricinus*	Poland	MZ146963
Bl._lanei	SW19	*I. ricinus*	Poland	MZ146975
Bl. americana	CM1296	*I. ricinus*	Poland	MZ146942
B. turcica	LB1573	*I. ricinus*	Poland	MZ146993
B._miyamotoi	SW98	*I. ricinus*	Poland	MZ146992

Table S4. Reference strains of the *Borreliaceae* spirochetes used for the comparison of the *flaB* gene fragment

Borreliaceae Species	Strain	Source	Country	Accession Number
Bl. afzelii	1A10N-IR	*I. ricinus* fed on *Vulpes vulpes*	Poland	KF422791
	9PD12L2-IR			KF918616
	34MR2062-FR			MG944963
	3A3F-IC	*I. canisuga* fed on *Vulpes vulpes*		HM802193
	15Z1L-IC			KF422794
	7N1N-IC			KF422789
	8N10L-IC			KF422796
	3N13L1-IC			KF918614
	22NM1779-LC			MG944961
	3A1F-IK	*Ixodes cf. kaiseri* fed on *Vulpes vulpes*		KF422787
	42DG2N10-IK			KF422865
	9M10-BL	*Vulpes vulpes* (blood)		KF422858
Bl. spielmanii	L32-EA	*Vulpes vulpes* (ear)		JF732881
	6MR2001-FR	*I. ricinus* fed on *Vulpes vulpes*		MG944976
	43MR2086-LK	*I. kaiseri* fed on *Vulpes vulpes*		MG944977
	22NM1790-LK			MT118981
	30NM1817-LK			MT118982
Bl. garinii	4M4L-IC	*I. canisuga* fed on *Vulpes vulpes*		HM802182
Bl. burgdorferi	3A2F-IC	*I. ricinus* fed on *Vulpes vulpes*		HM802191
Bl. valaisiana	22NM1795-LK	*I. kaiseri* fed on *Vulpes vulpes*		MT118979
Bl. bissettiae	22NM1781-LC	*I. canisuga* fed on *Vulpes vulpes*		MG944964
Bl. californiensis	23NM1807-LC	*I. canisuga* fed on *Vulpes vulpes*		MG944984
Bl. carolinensis	22NM1786-LC			MG944970
Bl._lanei	33NM1921-LC			MG944965
Bl. americana	29C4N2-IC			KF918619
Bl. lusitaniae	CM850-12	*I. ricinus*		MK604287
B. turcica	33NM1854-LC	*I. canisuga*		MG944997
B. miyamotoi	CM1132-12	*I. ricinus*		MK604458
Bl. finlandensis	SW180-12			MK604301
